# Stress, anxiety, and depression on traffic police officers: a data report

**DOI:** 10.3389/fpsyg.2025.1605684

**Published:** 2025-06-16

**Authors:** Carlos Ramos-Galarza, Jennifer Obregón, Fiamma Flores, Taysha Argoti, Diego D. Díaz-Guerra, Marena Hernández-Lugo, Yunier Broche-Pérez

**Affiliations:** ^1^Facultad de Salud y Bienestar, Carrera de Psicología Clínica, Pontificia Universidad Católica del Ecuador, Quito, Ecuador; ^2^Departamento de Psicología, Universidad Central Marta Abreu de Las Villas, Santa Clara, Cuba; ^3^Prisma Behavioral Center, West Palm Beach, FL, United States

**Keywords:** stress, anxiety, depression, traffic police, mental health, hormonal imbalance, cognitive alterations, psychosomatic symptoms

## 1 Introduction

Stress is the body's natural response to the demands of an individual's environment, comprising physical and emotional reactions (Sushmitha et al., 2023). It activates the “fight or flight” mechanism, allowing the body to prepare for real or perceived threats. Precisely, its physiological response relies on the release of cortisol, produced on the Hypothalamic-Pituitary-Adrenal (HPA) Axis and the Autonomic Nervous System activation, increasing functions such as heart rate and blood pressure (James et al., 2023; Sanchez-Castillo et al., 2023). Positive stress (eustress) is essential for performing daily life tasks. However, when it becomes chronic, it can disrupt normal physiological processes and potentially contribute to developing several health conditions, such as mood disorders and cognitive issues (Lu et al., 2021; Sushmitha et al., 2023).

Anxiety is an emotional state marked by sensations of unease, dread, and apprehension, typically arising in response to perceived threats or stressors. Although it represents a normal adaptive reaction to challenges, it is classified as a disorder when it becomes excessive, recurrent, and disrupts daily functioning (Ashipala and Shilunga, 2023). It can negatively affect the quality of life and daily functioning, associated with muscle tension, dizziness, restlessness, learning and memory impairments, and decreased social functioning (Abubakar et al., 2021; Ashipala and Shilunga, 2023).

Depression is one of the most globally prevalent mental health disorders, identified by chronic feelings of sadness, hopelessness, and diminished interest in previously enjoyable activities (McKeever et al., 2017). This condition carries significant consequences, including social isolation, family dysfunction, absenteeism, and decreased productivity in professional and academic environments. These effects are often linked to concentration difficulties, suicidal ideation, and an increased risk of unhealthy dietary habits and a sedentary lifestyle (Balatif, 2023; Sousa de Oliveira et al., 2024).

Stress, anxiety, and depression are common factors affecting traffic police officers. In our society, they routinely encounter reckless drivers who endanger their own lives and others (Yadav et al., 2022). Additional stressors include smoking, extended work hours, and lack of physical exercise, all of which have been linked to adverse outcomes on officers' mental health (Gullon-Scott and Longstaff, 2024).

Based on the previously described context, we propose this research to develop the database presented in this article. Our primary objective is to provide a perspective on how the mental health of traffic police officers is affected and how this type of data can inform future interventions to address these issues and improve the working conditions of this essential profession in our society.

## 2 Methods

### 2.1 Research design

This study has a cross-sectional design with a correlational scope, aiming to analyze the relationship between stress, depression, and anxiety with muscular, cognitive, and hormonal symptoms in traffic police officers. This research employed a quantitative approach with non-experimental methods. The data facilitated statistical analyses, including correlational, regression, and explanatory models, potentially employing structural equation modeling. All data were collected in accordance with ethical standards for research involving human participants. Participants were informed of their voluntary participation at all times, and data were gathered anonymously in an environment free from disturbances. The sample consisted of 146 traffic police officers representing the entire police unit in Quito, Ecuador. The socio-demographic data are presented in Table 1.

**Table 1:** Participant demographic data (*n* = 146).

**Demographic variable**	**Mean ±SD or *n* (%)**
**Gender**	
Female	42 (28.8%)
Male	104 (71.2%)
**Age**	33.58 ± 2.11
Female	33.17 ± 2.106
Male	33.75 ± 2.103 *t*_(144)_ = −1.51, *p* = 0.70
**Civil status**	
Single	39 (26.7%)
Married	67 (45.9%)
Divorced	9 (6.2%)
Separated	1 (0.7%)
Common-Law	28 (19.2%)
Widowed	2 (1.4%)
**Children**	
Yes	124 (84.9%)
No	22 (15.1%)
**No. of children**	
0	22 (15.1%)
1	30 (20.5%)
2	69 (47.3%)
3	16 (11.0%)
4	7 (4.8%)
5	2 (1.4%)
**Time worked (in years)**	9.75 ± 1.46
Female	9.43 ± 1.595
Male	9.88 ± 1.391 *t*_(144)_ = −1.68, *p* = 0.095
**Hours worked per day**	8.23 ± 0.797
Female	8.36 ± 1.122
Male	8.18 ± 0.619 *t*_(144)_ = 1.199, *p* = 0.232

### 2.2 Studio scenario

This research was conducted in a city with specific characteristics that should be considered when interpreting the data in other contexts with similar features. Quito, the capital of Ecuador, operates within a capitalist economic system. The majority of its population identifies as Catholic, and the average level of education is 11.75 years. Household incomes typically range between $750 and $ 1,300. The educational system is comparable to other capital cities worldwide, offering a full range of professional and postgraduate programs. Public transportation is regulated by metropolitan agency officers who oversee its management.

### 2.3 Measuring instruments

The instruments used to construct this database included the DASS-21 brief scale, which was employed to assess anxiety, stress, and depression (Lovibond and Lovibond, 1995). This questionnaire comprises 21 self-report items designed to measure these three variables in real-life contexts. Additionally, to evaluate cognitive, hormonal, and muscular symptoms, the research team developed a 12-item scale to assess these aspects. In the interpretation of the items from both questionnaires, the following response scale was applied: 0 = It did not happen to me, 1 = It happened a little or for a short period of time, 2 = It happened quite a bit or for a good part of the time, and 3 = It happened a lot or most of the time. The items of each scale are described below.

## 3 DASS-21 questionnaire

### 3.1 Items

- I had difficulty relieving tension.
- I noticed that my mouth was dry.
- I was unable to feel any positive emotion.
- I found it difficult to breathe.
- I had difficulty taking the initiative to do things.
- I overreacted in certain situations.
- I felt my hands trembling.
- I felt like I was expending a great amount of energy.
- I was worried about situations where I might panic or embarrass myself.
- I felt that nothing excited or motivated me.
- I felt restless.
- I found it difficult to relax.
- I felt sad and depressed.
- I could not tolerate anything that interfered with what I was doing.
- I felt on the verge of panic.
- I could not get excited about anything.
- I felt that I was worthless.
- I tended to get angry easily.
- I felt my heart pounding even though I had not engaged in physical activity.
- I felt fear for no apparent reason.
- I felt that life had no meaning.

## 4 Muscular, cognitive, and hormonal symptoms questionnaire

### 4.1 Items

- I have felt my body being very tense.
- I have experienced headaches.
- I feel back pain.
- I have difficulty retaining information and concentrating.
- I find it difficult to make decisions.
- I struggle to comprehend information.
- I have difficulty falling or staying asleep.
- I have noticed the appearance of acne, pimples, or other skin eruptions.
- I feel that I have lost or gained weight.
- I have experienced digestive problems.
- I have felt nausea or the urge to vomit.
- I frequently feel an upset stomach.

DASS-21 Scale demonstrated good internal consistency reliability in the current sample (21 items; Cronbach's α = 0.95), with a total-item correlation ranging from *r* = 0.29 −0.71, as well as the Muscular, Cognitive, and Hormonal Symptoms Questionnaire (12 items, α = 0.92), with a total-item correlation ranging from *r* = 0.28 −0.72. These psychometric properties indicate a high inter-item correlation and suggest that both Scales are reliable and valid instruments for assessing these symptoms in adult populations.

The instruments used in this research were employed in their original Spanish version. All the measurements underwent content validation by the expert research team. The process started with cognitive interviews to assess whether the participants understood the items well. Following this, the scale's psychometric properties were analyzed to proceed with the subsequent statistical analyses.

The questionnaires were administered in physical form in a distraction-free environment under the supervision of a research team member. Once the questionnaires were completed, the data were tabulated using Statistical Package for Social Sciences (SPSS) software for statistical analysis.

This database includes the following socio-demographic variables: gender, age, marital status, number of children, years of work experience, and daily hours.

## 5 Data description

Table 1 shows the demographic characteristics of the study participants, including key socio-demographic variables such as gender, age, civil status, number of children, and years of service in the police force. It also provides information on the participants' place of employment and the average hours worked per day. These variables offer a comprehensive overview of the sample, providing context for understanding the potential influence of personal and professional factors on the mental health of traffic police officers.

The statistical analysis of Table 2 presents the data from the DASS-21 questionnaire and the muscular, cognitive, and hormonal symptoms questionnaire. This table includes the frequency and percentage of responses for the 21 items from the DASS-21 scale and the 12 items from the additional questionnaire, offering a detailed overview of the participants' experiences and symptoms.

**Table 2:** Descriptive statistics: frequency and percentage.

**Item**	**0: It did not happen to me**	**1: It happened a little or for a short period of time**	**2: It happened quite a bit or for a good part of the time**	**3: It happened a lot or most of the time**
**DASS-21 questionnaire**				
1	39 (26.71%)	55 (37.67%)	32 (21.92%)	20 (13.70%)
2	51 (34.93%)	59 (40.41%)	24 (16.44%)	12 (8.22%)
3	69 (47.26%)	47 (32.20%)	17 (11.64%)	13 (8.90%)
4	92 (63.01%)	38 (26.03%)	10 (6.85%)	6 (4.11%)
5	58 (39.73%)	61 (41.78%)	17 (11.64%)	10 (6.85%)
6	34 (23.29%)	61 (41.78%)	38 (26.03%)	13 (8.90%)
7	69 (47.26%)	41 (28.09%)	27 (18.49%)	9 (6.16%)
8	29 (19.86%)	55 (37.67%)	44 (30.14%)	18 (12.33%)
9	39 (26.71%)	62 (42.47%)	32 (21.92%)	13 (8.90%)
10	64 (43.84)	44 (30.14%)	26 (17.81%)	12 (8.22%)
11	33 (22.60%)	76 (52.10%)	27 (18.50%)	10 (6.80%)
12	42 (28.77%)	64 (43.84%)	31 (21.23%)	9 (6.16%)
13	49 (33.56%)	56 (38.36%)	27 (18.49%)	14 (9.59%)
14	54 (36.99%)	58 (39.73%)	29 (19.86%)	5 (3.42%)
15	81 (55.5%)	37 (25.3%)	19 (13.0%)	9 (6.2%)
16	73 (50.00%)	46 (31.51%)	22 (15.17%)	5 (3.42%)
17	95 (65.07%)	24 (16.44%)	21 (14.38%)	6 (4.11%)
18	45 (30.82%)	50 (34.25%)	31 (21.23%)	20 (13.70%)
19	60 (41.10%)	61 (41.78%)	19 (13.01%)	6 (4.11%)
20	69 (47.3%)	57 (39.0%)	13 (8.9%)	7 (4.8%)
21	94 (64.38%)	26 (17.81%)	14 (9.59%)	14 (8.22%)
**Muscular, cognitive, and hormonal symptoms questionnaire**				
1	40 (27.40%)	60 (41.10%)	35 (23.97%)	11 (7.53%)
2	31 (21.23%)	60 (41.10%)	36 (24.66%)	19 (13.01%)
3	26 (17.81%)	56 (38.36%)	37 (25.34%)	27 (18.49%)
4	52 (35.62%)	58 (39.72%)	24 (16.44%)	12 (8.22%)
5	57 (39.04%)	61 (41.78%)	20 (13.70%)	8 (5.48%)
6	78 (53.42%)	42 (28.77%)	22 (15.07%)	4 (2.74%)
7	54 (36.99%)	46 (31.51%)	33 (22.60%)	13 (8.90%)
8	64 (43.84%)	47 (32.19%)	22 (15.07%)	13 (8.90%)
9	44 (30.14%)	58 (39.73%)	34 (23.29%)	10 (6.85%)
10	53 (36.30%)	47 (32.19%)	34 (23.29%)	12 (8.22%)
11	80 (54.8%)	33 (22.6%)	28 (19.2%)	5 (3.4%)
12	72 (49.32%)	41 (28.08%)	21 (14.38%)	12 (8.22%)

## 6 Limitations

In this database, it is important to consider three limitations that should influence the interpretation of statistical results. First, the geographical factor is crucial, as the data were collected from a single city in Latin America. This limitation highlights the need for further research to understand the role of stress, anxiety, and depression in the mental health of traffic police officers in different contexts. Second, the age range of the participants (30 to 38 years old) restricts the generalizability of the findings, limiting their applicability to traffic police officers outside this specific demographic group.

Third, the subjective nature of self-assessment may introduce bias in data collection. Some participants might be influenced by identity construction or the desire to present themselves in a socially desirable manner. These three factors should be considered in future studies within this research domain. New research efforts must account for these limitations to enhance understanding of this phenomenon.

## 7 Future research

Future research could employ a longitudinal design to monitor changes in stress, anxiety, and depression levels among traffic police officers over extended periods, offering insights into how these symptoms develop with prolonged exposure to occupational stressors. A multi-center or nationwide approach could enhance the generalizability of findings by encompassing diverse police units across varying work environments. Additionally, incorporating objective measures, such as cortisol levels to assess stress and clinical interviews to evaluate anxiety and depression, would strengthen the validity of the data and complement self-reported assessments. Moreover, future studies should consider implementing and evaluating mental health interventions, such as mindfulness-based programs or expanded access to psychological counseling, to determine their effectiveness in promoting well-being within this occupational group.

## Data Availability

The datasets presented in this study can be found in online repositories. The names of the repository/repositories and accession number(s) can be found in the article/supplementary material.
